# Monkeypox claims new victims: the outbreak in men who have sex with men

**DOI:** 10.1186/s40249-022-01007-6

**Published:** 2022-07-23

**Authors:** Xiaoning Liu, Zheng Zhu, Yun He, Jia Wen Lim, Bethany Lane, Hui Wang, Qiaoli Peng, Liqin Sun, Hongzhou Lu

**Affiliations:** 1 Department of Infectious Diseases, National Clinical Research Center for Infectious Diseases, The Third People’s Hospital of Shenzhen, Shenzhen, 518112 Guangdong China; 2 National Heart & Lung Institute, Faculty of Medicine, Imperial College London, London, UK; 3 School of Nursing, Fudan University, Shanghai, China; 4 Fudan University Centre for Evidence-Based Nursing: A Joanna Briggs Institute Centre of Excellence, Shanghai, China; 5 NYU Rory Meyers College of Nursing, New York University, New York, NY USA; 6 St Bartholomew’s Hospital, London, UK

**Keywords:** Monkeypox, Men have sex with men, Public health, Infectious diseases

## Abstract

Monkeypox has a very prominent regional epidemic. It has been confined to Western and Central African countries. Sporadic cases found in countries outside Africa generally have a history of sojourn in endemic areas. However, the recent multinational outbreak of monkeypox cases in Europe in early May 2022 has revealed a changing epidemiological trend, those confirmed cases had no sojourn history in endemic areas and with a high proportion of cases involving men who have sex with men (MSM). Among the MSM cases, many of them presented atypical clinical manifestations of monkeypox and with other sexually transmitted diseases co-infection. Combined with the high social interactivity in this community, there is likely a higher risk of monkeypox transmission in this population. Establishing an infectious disease surveillance system, maintaining highly vigilant regarding the transmission of monkeypox in MSM, and responding promptly are necessary and effective measures to contain the outbreak.

## Background

Monkeypox is a zoonotic disease caused by the monkeypox virus. With the eradication of smallpox in 1980 and subsequent discontinuation of the smallpox vaccination, monkeypox is emerging as the most significant pox virus affecting public health at present. Since the first human case was discovered in 1970, the disease has been confined to Western and Central Africa. However, since 2003, sporadic cases have been detected in countries outside of Africa, though these usually coincide with a history of exposure in endemic areas [1].

The recent multinational outbreak of monkeypox cases in Europe around the beginning of May 2022 has revealed a changing epidemiological trend [2]. As of June 22, 3413 confirmed cases have been reported in fifty countries and the number of cases is still rising [3]. These cases had no sojourn history in endemic areas and with a high proportion of cases involving men who have sex with men (MSM). Despite the fact that monkeypox virus was recently detected in the semen of confirmed cases in Italy, potential contamination of the specimens should be considered [4]. There is still no high-quality evidence that monkeypox can be transmitted sexually, highlighting a research gap that remains to be filled.

## Monkeypox characteristics in MSMs

Interestingly, many confirmed monkeypox cases in the MSM population do not exhibit the classic clinical manifestations. The most notable features are genital and perianal rashes, which often appear as the first symptoms. Furthermore, the morphology of the rash does not progress from maculopapular rash to blisters and pustules as in typical cases, instead, pustules have appeared before systemic symptoms (e.g., fever) in some cases [4–6]. The initial appearance of genital or perianal rash may imply that close physical contact during sexual intercourse acts as a possible transmission route. All reported cases of monkeypox in MSM had sexual exposure with or without using condoms prior to symptom onset [5, 6]. It is unclear whether condom use has a protective effect on monkeypox transmission. However, condom use is still strongly recommended to prevent other sexually transmitted diseases (STDs). Reported monkeypox cases in MSM include both HIV-positive cases with viral suppression and HIV-negative cases receiving pre-exposure prophylaxis, and many are diagnosed alongside other STDs. Coinfection with hepatitis A, B, or C is also common [4–8]. Detailed data on HIV and other STDs co-infection in confirmed cases is still absent. The World Health Organization (WHO) has subsequently developed a monkeypox case reporting form to capture this crucial information [9]. It is hoped that this data will improve understanding on the characteristics of MSM with monkeypox, to aid future prevention and treatment.

## Health threat of monkeypox in MSMs

Monkeypox is ordinarily a self-limiting disease. Clinical outcomes are related to the degree of viral exposure, patient's health status, and the nature of complications. Immunodeficiency, such as advanced or uncontrolled HIV infection may lead to more severe clinical manifestations. Since HIV patients usually have multiple comorbidities, such as hepatitis, tuberculosis, and/or other STDs, this can cause additional complications and complicate treatment. In addition, due to similarities in the presentations of the monkeypox rash and some STDs, misdiagnosis may be more common in the MSM population than in the general population. It was reported that some cases were misdiagnosed as herpes simplex virus (HSV) or varicella-zoster virus (VZV) infection in this outbreak [5], resulting in late detection and management, thus increasing the risk of community transmission. Furthermore, the high social interactivity in MSM contributes to a high risk of monkeypox transmission in this population.

## Recommendations for the management of monkeypox in MSMs

A well-developed infectious disease surveillance system facilitates early detection of diseases and contact tracing. Some countries, such as United Kingdom and the Republic of Korea, have listed monkeypox as a statutory notifiable disease. Healthcare workers should be highly vigilant about monkeypox transmission in MSM. It is necessary to consider the diagnosis of monkeypox in MSM patients with a typical rash and risky sexual behaviour, especially in those with a history of sexual contact at the site of the disease outbreak. Patients having a sexual history in MSM should be actively screened for HIV and other STD infections. For those with HIV co-infection, antiretroviral therapy and viral load monitoring should be provided as an urgent public health priority. Finally, although current monkeypox outbreaks are mainly in the MSM populations, it is crucial to avoid stigmatisation. Effective communication and community engagement are paramount to ending the monkeypox outbreak.

## Conclusions

The emerging monkeypox outbreak in non-endemic areas featured by absence of sojourn history in endemic areas and high proportion of MSMs, has garnered international interest. There is currently no high-quality evidence that monkeypox can be transmitted sexually, exposing an unfilled research gap. The WHO has announced great concern over the progress of the outbreak. Establishing an infectious disease surveillance system, maintaining highly vigilant regarding the transmission of monkeypox in MSM, and intervening early are necessary and effective measures to contain the outbreak.

## Data Availability

Not applicable.

## Abbreviations

MSM: Men who have sex with men
STDs: Sexually transmitted diseases
HIV: Human immunodeficiency virus
WHO: World Health Organization
HSV: Herpes simplex virus
VZV: Varicella-zoster virus
